# The Effects of Exercise Regimens on Irisin Levels in Obese Rats Model: Comparing High-Intensity Intermittent with Continuous Moderate-Intensity Training

**DOI:** 10.1155/2018/4708287

**Published:** 2018-12-27

**Authors:** Neng Tine Kartinah, Imelda Rosalyn Sianipar, Nafi'ah, Rabia

**Affiliations:** ^1^Department of Medical Physiology, Faculty of Medicine, Universitas Indonesia, Jakarta 10430, Indonesia; ^2^Master Program in Biomedical Sciences, Faculty of Medicine, Universitas Indonesia, Jakarta 10430, Indonesia

## Abstract

**Background:**

Recently, high-intensity intermittent training (HIIT) appears to have the same beneficial effects or even superior to those of continuous moderate-intensity training (CMIT) on body fat mass reduction. Exercise may induce myokine secretion such as irisin, which plays a role as a mediator of beiging process, and thus might contribute as treatment of obesity. However, the effects of those exercise formulas on irisin level changes as beiging agent are not known. In addition, metabolic states may affect the irisin responses to those exercise formulas. Therefore, this study was aimed to determine the different effects of exercises using HIIT and CMIT on circulating and tissue irisin levels in normal and abnormal metabolic conditions (obese).

**Methods:**

Sixteen male Sprague-Dawley rats (8 weeks of age) were randomized to 4 groups according to training regimens (HIIT and CMIT) and metabolic conditions (normal and abnormal/obese). The groups are (1) HIIT on normal metabolic (n=4), (2) CMIT on normal metabolic (n=4), (3) HIIT on abnormal metabolic (n=4), and (4) CMIT on abnormal metabolic (n=4). Abnormal metabolic condition was induced with high fat diet (19% fat) for 8 weeks in obese rats. Irisin levels in serum, skeletal muscle, and white adipose tissue were evaluated by ELISA.

**Results:**

Serum irisin levels were shown significantly higher in normal metabolic compared to abnormal metabolic condition (P<0.001). The effect of interaction between metabolic condition and exercise formula was found (P<0.01) on adipose irisin levels. The effect of HIIT was shown significantly more effective on adipose irisin levels, compared with CMIT in abnormal metabolic conditions. However, no significant differences of skeletal muscle irisin levels were found in both normal and abnormal metabolic subjects (P>0.05). Regarding exercise formula, no different effects were found between HIIT and CMIT on skeletal muscle irisin levels in both metabolic conditions (P>0.05). The similar findings were observed in serum irisin levels (P>0.05).

**Conclusions:**

The exercise effects in abnormal metabolic condition might be more adaptable in maintaining the irisin levels in skeletal muscle and induce the irisin uptake from circulation into adipose tissue. In addition, HIIT might be more involved to induce irisin uptake into adipose tissue; thus it might have the significant role in beiging process. However, further research about how the HIIT formula affects the regulation mechanisms of irisin uptake into adipose tissue is still warranted.

## 1. Introduction

Exercise has been proposed as one of the approaches to combat obesity. A study conducted by Hankinson et al. (2010) showed that exercise significantly decreased body weight in obese subjects [1]. Experiments using rodents have revealed that exercise plays a role in adipose tissue browning which is the transitional changes of characteristics from white adipose tissue to beige adipose tissue. Beiging/browning process has a potential role as a protection against obesity, because it may increase thermogenesis activity which consequently results in the increase of lipid catabolism and releases heat and thus prevents excessive accumulation of fat [2, 3].

Bostrom et al. (2012) identified a novel exercise-induced myokine which has a role as a mediator of beiging process, irisin [4]. Exercise as stimuli can induce irisin secretion from skeletal muscle through different mechanisms. PGC-1*α*, as a master regulator of irisin secretion, is a factor which is being upregulated during and after exercise in skeletal muscle. Irisin then is secreted from skeletal muscle into circulation by proteolytical cleavage of FNDC5 transmembrane protein. It has been suggested that intracellular ATP depletion in skeletal muscle also plays a contributing role in affecting irisin secretion [5]. After secretion from skeletal muscle, irisin acts to express UCP-1 in adipose tissue which leads to browning of white adipose tissue [5].

However, the effect of exercise on serum irisin level showed the contradicting results. In human studies, Norheim et al. (2014) suggested the decrease of irisin level after 12-week combination exercises (endurance and strength exercises) in sedentary individuals at age of 40-65 years. The similar findings were observed in the study conducted by Tsuchiya et al. (2015) using twenty healthy males after 4-week sprint training. In contrast, Hecksteden et al. (2013) reported that 26-week exercise (aerobic versus strength endurance training) could not result in irisin level changes in healthy subjects. Another study conducted by Moienneia et al. (2016) reported a significant decrease of irisin level after high-intensity resistance training, but not after low-intensity resistance training in sedentary young women. Other findings were reported by Kim et al. (2015) that 8-week exercises (aerobic and resistance training) increased circulating irisin levels. Norheim et al. (2014) also reported an increase of skeletal muscle FNDC5 mRNA levels following 12-week intervention of combined endurance and strength training. The contradicting results of irisin responses induced by exercise might be resulted from the difference of exercise formula (dosage). Exercise formula included frequency, intensity, type, and time should be considered to improve the optimal outcome exercise [6–11].

The study conducted by Shin* et al*. using obese rats recommended continuous moderate-intensity training (CIMT) to treat obesity [12]. In addition, Ahmed et al. (2013) suggested that 1-hour moderate-intensity exercise could decrease body fat mass in high fat diet induced rats [13]. However, high-intensity intermittent training (HIIT) recently gained more interests [14]. Many studies showed that HIIT could decrease body weight, as efficient as CMIT. In addition, Stephen et al. (2011) reported that HIIT decreased body and subcutaneous fat mass [14]. Moreover, Jamali et al. (2016) showed that in obese rats HIIT could decrease body weight of high fat diet-induced rats [15]. Another study conducted by Tiano et al. using obese mice showed that forced treadmill exercise gave the strongest skeletal muscle FNDC5 (irisin precursor) response, compared with forced swimming and free running on the wheel [16]. In addition, Rocha-Rodrigues et al. (2016) have shown that endurance training (treadmill) increased skeletal muscle FNDC5 protein expression; thus it seems to contribute to the “brown-like” phenotype in white adipose tissue from high fat diet fed rats [17].

Based on the above explanation, the effects of HIIT and CMIT on irisin level changes are not known, yet. Besides the exercise formula, the metabolic condition also may affect the irisin responses. Irisin is a molecule which plays a big role in maintaining metabolic homeostasis. As a metabolic regulator, irisin levels might be varied or fluctuating based on metabolic states (demands). Huh et al. (2015) reported no significantly different effects of exercises on irisin level between healthy subjects and those with metabolic syndrome. In contrast, the study conducted by Tibana et al. (2016) showed the differences of irisin responses to exercise between nonobese and obese subjects [7, 8, 10, 18]. From the above explanations, the optimal exercise formulas to induce irisin secretion have not been known yet. In addition, metabolic states may contribute to irisin responses to exercise formula. Therefore, this study was aimed to determine the different effects of exercises using HIIT and CMIT on circulating and tissue irisin levels in normal and abnormal metabolic conditions. Abnormal metabolic conditions were shown in obese rats fed with high fat diet.

## 2. Materials and Methods

### 2.1. Study and Design

All experiments were approved by the Ethical Committee of Faculty of Medicine, Universitas Indonesia. 16 male Sprague-Dawley rats (V-Stem, Bogor), 8 weeks old, were housed in proportion 3 rats per cage with a 12h:12h light/dark cycle.

### 2.2. Animal

Sixteen male Sprague-Dawley rats (8 weeks of age) were randomized to 4 groups according to training regimens (HIIT and CMIT) and metabolic conditions (normal and abnormal/obese). The groups are (1) HIIT on normal metabolic (n=4), (2) CMIT on normal metabolic (n=4), (3) HIIT on abnormal metabolic (n=4), and (4) CMIT on abnormal metabolic (n=4). At the beginning, rats were randomly assigned, into two groups: (1) normal metabolic group, involving 8 rats fed with standard laboratory pellet (consisting of 6% fat), and (2) abnormal metabolic group fed with high fat (HF), involving the other 8 rats fed with high fat pellet (consisting of 19% fat) [19, 20]. After 10 weeks, group fed with high fat diet presented Lee Index > 310 [21] which were categorized as obese (data were not shown). Normal groups represented normal metabolic condition, but obese groups fed with high fat diet represented abnormal metabolic condition. After the first 10 weeks, each group was subdivided into 2 groups (n=4 per group): (1) high-intensity intermittent training (HIIT) group, and (2) continuous moderate-intensity training (CMIT) group. The diet was continuously provided during exercise training.

Sample size was determined using* Research Equation Method*, where E is the total number of animals – total number of groups. The value of E should be lied between 10 and 20. Any sample size, which keeps E between 10 and 20, should be considered as an adequate. Total numbers of groups in this study were 4 groups. If each group consisted of 4 animals, total number of animals used in this study were 16, then E will be valued 12 and hence can be considered as adequate sample size [22].

### 2.3. Exercise Protocol

Before starting exercise training, all rats underwent an adaptation period in order to minimize the potential stress from equipment. Exercise training was conducted 5 days per week for 8 weeks, while diet feeding was still provided simultaneously. HIIT protocol (Table 1) was adopted from Jamali et al., 2016, which involved running activity on an animal treadmill. The protocol included 5 minutes of warming up and 5 minutes of cooling down at 10 meters/minute. After warming up, exercise session started with 5 repetitions of 30 seconds at 29 meters/minute along with one-minute active rest interval. The intensity including repetition and speed increases per week. The active rest was the continuation of running on treadmill but at 13 meters/minute.

**Figure 1:**
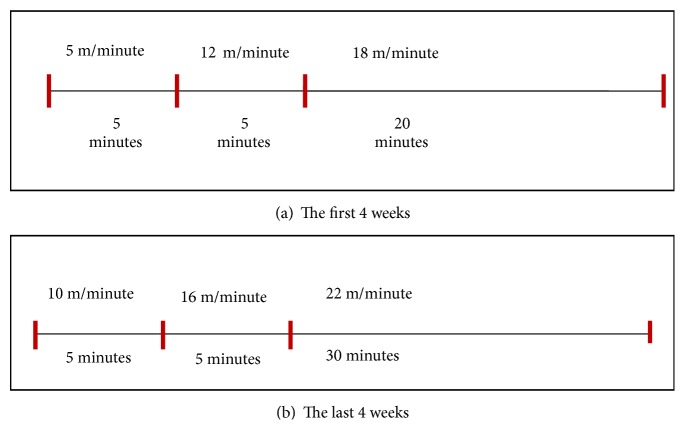
CMIT protocols.

CMIT protocol (Figure 1) adopted from Ki Ok Shin et al., 2015, consisted of 5 m/min for 5 minutes, 12m/min for 5 minutes, and 18m/min for 20 minutes for the first four weeks. For the last 4 weeks, protocol consisted of 10 m/minute for 5 minutes, 16 m/minute for another 5 minutes, and 22 m/minute for the last 30 minutes.

### 2.4. Sampling

Forty-eight hours after the last session of exercise training, rats were anesthetized with Ketamine (0.05 ml/kg) and Xylazine (0.01 ml/kg) injected intraperitoneally. Blood was collected intraventricularly in 3 mL sampling tube. Serum was obtained after a 10 min centrifugation at 3000 rpm. Subcutaneous adipose and gastrocnemius tissue were collected. Serum and tissues were immediately stored at -80°C for further analysis.

### 2.5. Measurement of Irisin Concentration

The tissue samples (90-100 mg) were homogenized with 1000 *μ*L ice-cold PBS using ultra-turrax homogenizer. Homogenates were centrifuged for 20 minutes at 12,000* g *at 4°C. Supernatants were removed and aliquots were stored in -80.C. Protein contents of the homogenates were quantified using a Thermo fisher Bradford Assay.

Quantitative measurement of irisin in rat serum and tissue homogenate samples was performed using a commercial enzyme-linked immunosorbent assay (ELISA) kit (Phoenix Pharmaceuticals, Inc.; EK-067-29) according to the manufacturer's instructions. Absorbance from each sample was measured in duplicate using a microplate reader at a wavelength of 450 nm. For tissue homogenate samples, irisin concentration data (ng/mg) were presented as a ratio between irisin concentration (ng/mL) and total protein content of homogenates (mg/mL).

### 2.6. Statistical Analysis

All measurement data are expressed as mean ± standard error mean (SEM) and analyzed using two-way analysis of variance (ANOVA). A* P* value < 0.05 was considered statistically significant. All statistical analyses were performed using the statistical software SPSS version 21.0.

## 3. Results

### 3.1. The Effects of Metabolic Condition and Exercise Formula on Skeletal Muscle Irisin Levels

**Figure 2:**
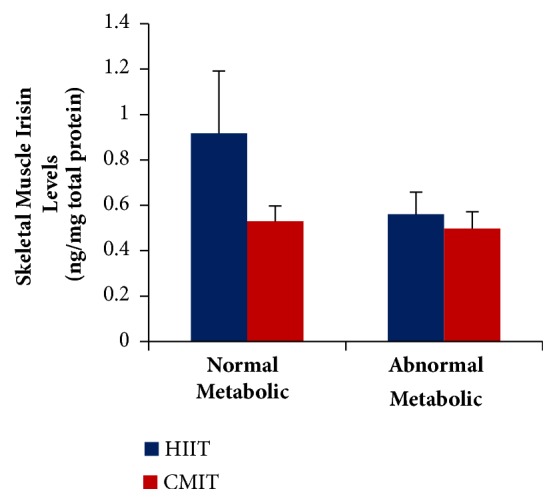
**The effects of metabolic condition and exercise formula on skeletal muscle irisin level.** Values are presented as Mean±SEM. Data were analyzed using* two-way* ANOVA.** HIIT**, high-intensity intermittent training;** CMIT**, continuous moderate-intensity training.

It was observed that abnormal metabolic groups presented lower skeletal muscle irisin levels compared to normal metabolic groups. However, the differences were not statistically significant (F(1.59);* P*> 0.05). It showed that exercise could result in response changes of irisin levels in abnormal metabolic condition; thus we found no significant differences of irisin levels compared with normal metabolic condition. This study also showed no differences between HIIT and CMIT on skeletal muscle irisin levels either in normal or in abnormal metabolic condition (F(2.17);* P*>0.05). Therefore, both HIIT and CMIT resulted in same responses on skeletal muscle irisin levels either in normal or in abnormal metabolic condition (Figure 2).

### 3.2. The Effects of Metabolic Condition and Exercise Formula on Serum Irisin Levels

**Figure 3:**
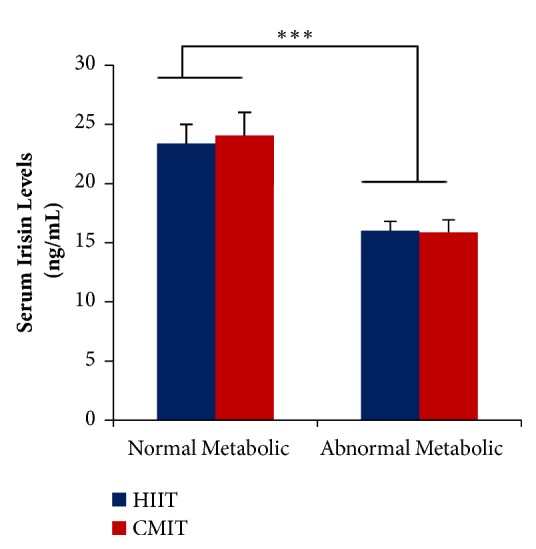
**The effects of metabolic condition and exercise formula on serum irisin levels.** *∗∗∗*P < 0.001. Values are presented as Mean±SEM. Data were analyzed using* two-way* ANOVA.** HIIT**, high-intensity intermittent training;** CMIT**, continuous moderate-intensity training.

Both HIIT and CMIT have no different effects on serum irisin levels (F(0.035);* P*>0.05) in both normal metabolic and abnormal metabolic groups. These findings suggest that both HIIT and CMIT had the same effects on serum irisin level changes. However, the effects of metabolic conditions were found statistically significant on serum irisin levels. Abnormal metabolic groups presented significantly lower serum irisin level when compared to control group (F(29.48);* P*<0.001). It showed that obese condition might play a significant role in lowering irisin levels in serum (Figure 3).

### 3.3. The Effects of Metabolic Condition and Exercise Formula on Adipose Irisin Levels

**Figure 4:**
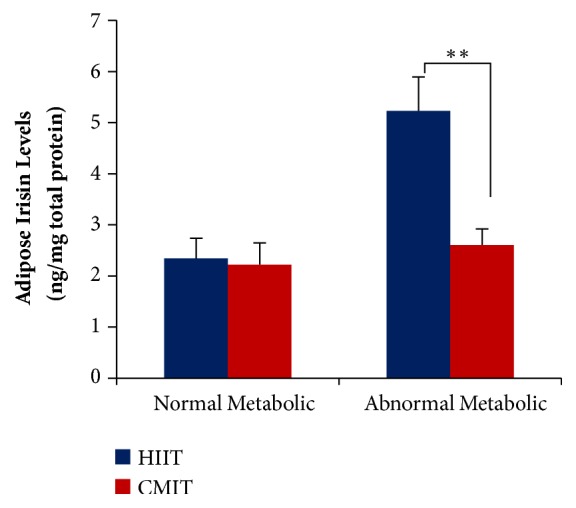
**The effects of metabolic condition and exercise formula on adipose irisin levels.** *∗∗*P < 0.01. Values are presented as Mean±SEM. Data were analyzed using* two-way* ANOVA.** HIIT**, high-intensity intermittent training;** CMIT**, continuous moderate-intensity training.

Both HIIT and CMIT had the significant effects on adipose irisin levels in abnormal metabolic conditions (F(15.67);* P*<0.01). However, HIIT was significantly effective in elevating the adipose irisin levels compared with CMIT. In contrast, no significant differences of both exercises were found in normal metabolic conditions (F(0.03);* P*> 0.05). The interaction effects between metabolic condition and exercise suggested that irisin responses in adipose tissue which resulted from exercise depend on the metabolic conditions (Figure 4).

## 4. Discussion

The results of the present study have revealed that no significant differences of skeletal muscle irisin levels were found following training in both normal and abnormal metabolic conditions. Those findings were in line with the findings on serum, which showed no different effect between HIIT and CMIT on serum irisin level. However, abnormal metabolic groups presented lower serum irisin level compared with normal metabolic group following both training regimens. In contrary, in adipose tissue, HIIT-abnormal metabolic group showed the higher irisin levels compared with CMIT group. Thus, HIIT might be more involved in increasing adipose irisin levels in abnormal metabolic condition, but the effects on skeletal irisin were not much different from those of CMIT, in both metabolic conditions.

In fact, abnormal metabolic condition might decrease the irisin levels in skeletal muscle. It was corroborated with the study conducted by de Macedo et al. (2017) which suggested that obese condition significantly decreased FNDC5 and irisin in skeletal muscle of mice [23]. This phenomenon might result from the increase of myostatin (MSTN) expression in skeletal muscle that plays a role in phosphorylation and activation of mothers against decapentaplegic homolog 3 (SMAD3) in obese subjects. SMAD3 functions to inhibit the production of irisin. In skeletal muscles, SMAD3 binds to promoter region of FNDC5 gene and Ppargc1a and repressed the expression of those genes [5]. However, in this study, the exercises could compensate the negative effect of obesity on irisin levels; thus no significant differences of skeletal muscle irisin levels were found between normal and abnormal metabolic condition. Our findings were in line with study conducted by Tiano et al. in high fat diet mice which suggested that exercise intervention (free running wheels, treadmill, and countercurrent swim tank) for 2-3 weeks could elevate the irisin levels in skeletal muscle. Exercise can increase irisin secretion in skeletal muscle by elevating the expression and activation of PGC-1*α*. PGC-1*α* has a function as main regulator of irisin secretion. In addition with upregulation of PGC-1*α*, depletion of intracellular ATP of skeletal muscles following exercise may induce synthesis of FNDC5 and irisin. In abnormal metabolic condition, exercise may decrease SMAD3 levels and thus contributes to elevation of irisin secretion [16]. Another study suggested that, in abnormal metabolic condition, exercise could increase irisin levels in skeletal muscle [24]. Therefore, adaptation responses of exercise in abnormal metabolic condition might be greater compared with normal metabolic condition. In addition, both HIIT and CMIT had the same impact on irisin level changes in skeletal muscle.

Irisin secretion from skeletal muscle had a greater contribution to circulating irisin compared with secretion from adipose tissue [5]. Study conducted in mice showed that secretion of irisin from skeletal muscle represents 72% of circulating irisin and the other 28% from adipose tissue secretion [5]. However, our study found that abnormal metabolic groups presented lower serum irisin level compared with normal metabolic group following training. These findings were not in accordance with the findings shown in skeletal muscle, which suggested no differences of irisin level between normal and abnormal metabolic condition. Our findings were not in line with study conducted by Huh et al. which reported that changes in circulating irisin levels were not significantly different between healthy subject and subject with metabolic syndromes, following high-intensity intermittent exercise (HIIE), continuous moderate exercise (CME), and resistance exercise (RE). In study conducted by Huh et al., irisin responses were measured 1 hour after exercise cessation, which is different from our study. It might be underlying the contradicting results found in our study [25]. Another findings by Yang et al. also reported that groups fed with high fat diet and engaged with swimming exercise for 16 weeks resulted in higher circulating irisin compared with those fed with high fat diet and no exercise [26]. Those contradicting results might result from the metabolic condition. Study conducted by Yang et al. provided high fat diet in normal rats, while our study provided high fat diet for established obese rats. Moreover, the duration of exercise intervention also might contribute to different results.

Besides, study conducted by Yang et al. did not evaluate irisin levels in adipose tissue while in our study, we found the higher irisin levels of adipose tissue in abnormal metabolic group, especially following HIIT. The underlying mechanisms of this phenomenon suggest that lower irisin levels in circulation and higher irisin levels in adipose tissue of abnormal metabolic subjects indicate that irisin uptakes from circulation into adipose tissue could be involved. Therefore, we assumed that irisin uptakes into adipose tissue were increased in an abnormal metabolic condition following training. The mechanisms of exercise in affecting irisin uptakes are still unknown, but Chen et al. assumed the mechanisms of irisin uptake involving exocytosis process [26, 27].

Our assumptions were corroborated with the study conducted by Chen et al. which suggested that, under physiological stresses, irisin transfer from circulation into tissue was increased [27]. As we know, exercise is one of the conditions under physiological stresses. Concerning exercise formula, HIIT seems to be more effective in elevating adipose irisin levels compared to CMIT in obese group, because HIIT might induce greater stress responses than CMIT. Thus, these might contribute to an increase in physiological stress, which consequently resulted in increased irisin uptake into adipose tissue. However, further researches are still needed to investigate the role of exercise in affecting irisin uptake (i.e., exocytosis mechanism) in adipose tissue. In addition, excessive lipid accumulation could result in metabolic stress in obese condition [28]. Thus, these also might contribute to an increase in physiological stress, which consequently resulted in increased irisin uptake into adipose tissue.

Irisin uptake in adipose tissue could mediate the protective effect from further complication which resulted from excessive lipid accumulation in adipose tissue [5]. Irisin in adipose tissue might play a role in inducing beiging process which involved an increase of lipid oxidation, glucose uptake from circulation, and thermogenesis activity and thus could present a potential therapeutic intervention for obesity [5]. Concerning exercise formula, HIIT may induce the beiging process of white adipose tissue in high fat diet fed mice more effectively compared with CMIT [29].

## 5. Conclusions

The exercise formula effects in abnormal metabolic condition might be more adaptable in maintaining the irisin levels in skeletal muscle and induce the irisin uptake from circulation into adipose tissue. In addition, HIIT might be more involved to induce irisin uptake into adipose tissue; thus it might have the major role in beiging process. However, further research about how the HIIT formula affects the regulation mechanisms of irisin uptake into adipose tissue is still warranted.

## Figures and Tables

**Table 1:** HIIT protocols.

**Week**	**Frequency**	**Warm-up**	**Duration**	**Intensity**	**Cooling down**
**1**	5 days/week	5 minutes (10 m/min)	16.5 minutes	5 rep. 30 s. 29 m/minute. Active Rest: 13m/minute. 1 minute.	5 minutes (10 m/min)

**2**	5 days/week	5 minutes (10 m/min)	18 minutes	6 rep. 30 s. 30 m/minute. Active Rest: 13m/minute. 1 minute.	5 minutes (10 m/min)

**3**	5 days/week	5 minutes (10 m/min)	19.5 minutes	7 rep. 30 s. 31 m/menit Istirahataktif 13 m/menit. 1 menit.	5 minutes (10 m/min))

**4**	5 days/week	5 minutes (10 m/min)	21 minutes	8 rep. 30 s. 32 m/minute. Active Rest: 13m/minute. 1 minute.	5 minutes (10 m/min)

**5**	5 days/week	5 minutes (10 m/min)	22.5 minutes	9 rep. 30 s. 33 m/minute. Active Rest: 13m/minute. 1 minute.	5 minutes (10 m/min)

**6**	5 days/week	5 minutes (10 m/min)	24 minutes	10 rep. 30 s. 34 m/minute. Active Rest: 13m/minute. 1 minute.	5 minutes (10 m/min)

**7**	5 days/week	5 minutes (10 m/min)	25.5 minutes	11 rep. 30 s. 35 m/minute. Active Rest: 13m/minute. 1 minute.	5 minutes (10 m/min)

**8**	5 days/week	5 minutes (10 m/min)	27 minutes	12 rep. 30 s. 36 m/minute. Active Rest: 13m/minute. 1 minute.	5 minutes (10 m/min)

## Data Availability

The raw (excel) data used to support the findings of this study are available from the corresponding author upon request.
